# The complex role of SIRT7 in p53 stabilization: nucleophosmin joins the debate

**DOI:** 10.1080/23723556.2021.1896349

**Published:** 2021-03-31

**Authors:** Poonam Kumari, Shahriar Tarighi, Thomas Braun, Alessandro Ianni

**Affiliations:** Department of Cardiac Development and Remodeling, Max-Planck-Institute for Heart and Lung Research, Bad Nauheim, Germany

**Keywords:** SIRT7, nucleophosmin, p53, nucleolus, ultraviolet irradiation

## Abstract

Release of nucleophosmin (NPM) from nucleoli following stress promotes rapid stabilization of the tumor suppressor p53 (TP53, best known as p53). Nucleoplasmic NPM binds to the ubiquitin ligase mouse double minute 2 (MDM2) and prevents MDM2-dependent p53 degradation. We recently demonstrated that sirtuin 7 (SIRT7) activates this pathway by directly deacetylating NPM following ultraviolet irradiation, indicating tumor-suppressive functions of SIRT7.

## Author´s view

The transcription factor p53 (TP53 in humans and Trp53 in mice) acts as a potent tumor suppressor by orchestrating a complex network of signaling pathways that counteract tumorigenesis. p53 limits the expansion of cells that have acquired potential tumorigenic mutations either by promoting cell cycle arrest and DNA repair or by inducing apoptosis of highly damaged cells.^1^ In addition, p53 controls numerous mechanisms allowing adaptation of cells to adverse conditions such as stimulation of anti-oxidant defenses, control of metabolism and autophagy, and several others.^1^ Consistent with its tumor-suppressive functions, inactivation of *Trp53* increases spontaneous and carcinogens-induced tumorigenesis in mice. In fact, inactivating mutations of *TP53* are present in more than 50% of human cancers. Most *TP53* mutations arise at different stages of cancer development, while germline homozygous *TP53* mutations were also discovered in humans and cause a rare cancer predisposition known as Li-Fraumeni syndrome.^1^

Under physiological conditions, cells maintain low p53 protein levels to avoid adverse effects of p53 activity. The E3 ubiquitin ligase MDM2 (mouse double minute 2) is a potent inhibitor of p53. MDM2 binds directly to p53, promotes p53 ubiquitination and subsequent degradation by the proteasome, thus destabilizing the protein. Rapid activation of the p53 response following genotoxic insults is imperative to efficiently switch on tumor-suppressive functions of p53.^2^ Compared to the initiation of *p53* gene expression, which takes relatively long to robustly increase p53 protein levels, inhibition of MDM2 is very fast and provides a very efficient means to enhance p53 levels. Post-translational modifications (PTMs) of MDM2 and also of p53 contribute to the disruption of MDM2-p53 complex. However, PTMs alone are not sufficient to achieve a robust elevation of p53 protein levels under prolonged stress exposure.^2^

In the last years, the nucleolus has been recognized as a critical compartment, actively promoting p53 stabilization under stress by interfering with MDM2-p53 interaction. The nucleolus is not only involved in ribosome biogenesis but rather acts as a key sensor for diverse stress stimuli that orchestrates cellular stress responses.^3^ In response to different stress signals, the nucleolus undergoes a dramatic reorganization, favoring the release of nucleolar- and ribosomes-associated proteins, which allows interactions with downstream targets that reside in the nucleoplasm and/or in the cytoplasm.^3^ This phenomenon is referred to as nucleolar stress response (NSR).^3^ Release of nucleophosmin (NPM) from the nucleolus is a hallmark of the NSR and essential to promote p53 stabilization. After release from nucleoli, NPM binds to MDM2 and prevents the interaction of MDM2 with p53, thus inhibiting MDM2-mediated degradation of p53. The molecular mechanisms governing NPM translocation from the nucleolus and its interaction with MDM2 have remained largely uncharacterized until now.^4^ We recently demonstrated that deacetylation of NPM by the NAD^+^-dependent histone/protein deacetylase SIRT7 plays a fundamental role in stabilizing p53 in response to ultraviolet (UV)-induced genotoxic stress by activating this pathway.^4^

**Figure 1.**
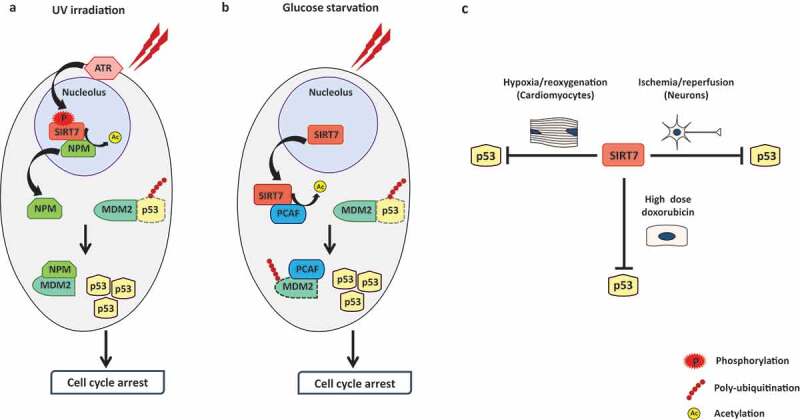
Mechanisms of SIRT7-dependent p53 stabilization in response to stress. **A**. Ultraviolet (UV) irradiation increases the catalytic activity of sirtuin 7 (SIRT7) by promoting SIRT7 phosphorylation by Ataxia telangiectasia mutated and Rad3 related kinase (ATR). Activated SIRT7 deacetylates nucleophosmin (NPM), which leaves nucleoli and binds to the ubiquitin ligase murine double minute 2 (MDM2), thereby inhibiting MDM2-dependent p53 ubiquitination and degradation. **B**. Glucose starvation facilitates release of SIRT7 from nucleoli, allowing interaction with the acetyltransferase p300/CBP-associated factor (PCAF). SIRT7 deacetylates PCAF, which subsequently binds to MDM2, thus promoting MDM2 ubiquitination and destabilization, resulting in accumulation of p53. **C**. SIRT7 inhibits p53 accumulation following exposure to high doses of doxorubicin, in neurons exposed to ischemia/reperfusion injury and in cardiomyocytes following hypoxia/reoxygenation-induced stress

SIRT7 belongs to the family of mammalian Sirtuins, which consists of seven members (SIRT1-SIRT7). These molecules act as key players in promoting cellular adaptation to environmental stressors by activating a complex network of cellular stress responses. SIRT7 is the only member of the sirtuin family that resides in the nucleolus.^5^ In our recent study, we demonstrated that under physiological conditions SIRT7 and NPM form a molecular complex in the nucleolus. However, exposure to UV-irradiation dramatically increases the catalytic activity of SIRT7 due to phosphorylation by the ATR kinase (Ataxia telangiectasia mutated and Rad3 related), a major activator of the DNA damage response (DDR).^4^ The enhanced enzymatic activity enables SIRT7 to deacetylate NPM at two distinct residues: lysine 27 and lysine 54. Deacetylated NPM leaves the nucleoli, strongly binds to MDM2 and inhibits MDM2-mediated ubiquitination and degradation of p53, thus promoting p53 accumulation. As a net result, SIRT7 depleted cells display blunted stabilization of p53 following UV-irradiation and are less prone to p53-induced cell cycle arrest as compared to control cells (Figure 1a). ^4^

SIRT7 seems to employ different strategies to stabilize p53, depending on the type of the stressor. In a recent study, it was shown that under glucose starvation SIRT7 elevates p53 protein levels by destabilizing MDM2.^6^ Mechanistically, SIRT7 deacetylates the acetyltransferase PCAF (p300/CBP-associated factor) thereby promoting binding of PCAF to MDM2, which will eventually destabilize MDM2 (Figure 1b). ^6^ Interestingly, we found that SIRT7 has no impact on MDM2 stability following UV-irradiation, indicating that SIRT7 inhibits MDM2 either by deacetylating NPM or PCAF.^4,6^ In sharp contrast, SIRT7 does not increase but diminish p53 levels following treatment with high doses of the potent anticancer drug doxorubicin, probably by attenuating the DDR, independent of NPM deacetylation.^4,7^ Moreover, SIRT7 destabilizes p53 in neurons exposed to ischemia/reperfusion injury and in cardiomyocytes subjected to hypoxia/reoxygenation injury, although the underlying mechanisms remain largely uncharacterized (Figure 1c). ^8,9^ The capacity of SIRT7 to directly deacetylate p53 and repress its transactivation activity adds another layer of complexity to SIRT7-mediated control of p53. Under specific stress conditions, SIRT7 is able to directly deacetylate p53 in some cell types but not in others.^6,7,9,10^ The context-dependent function of SIRT7 in the control of p53 activities is complex and warrants further research. Further understanding of the molecular mechanisms that control the SIRT7-p53 axis in response to specific stressors may offer new opportunities for the development of novel anti-cancer therapies.
